# Exploring of spectrum beta lactamase producing multidrug-resistant *Salmonella enterica* serovars in goat meat markets of Bangladesh

**DOI:** 10.1016/j.vas.2024.100367

**Published:** 2024-06-02

**Authors:** Jarin Al Naser, Hemayet Hossain, Md. Shahidur Rahman Chowdhury, Nasrin Akter Liza, Rayhan Mahmud Lasker, Asikur Rahman, Md. Ariful Haque, Md. Mukter Hossain, Md. Mahfujur Rahman

**Affiliations:** a Department of Medicine, Faculty of Veterinary, Animal and Biomedical Sciences, Sylhet Agricultural University, Sylhet-3100, Bangladesh; b Department of Anatomy and Histology, Faculty of Veterinary, Animal and Biomedical Sciences, Sylhet Agricultural University, Sylhet-3100, Bangladesh

**Keywords:** Goat meat, Multi-Drug Resistance, One health approach, Salmonella enterica serovars, Zoonotic transmission

## Abstract

The emergence of *Salmonella enterica* serovars that produce extended-spectrum beta-lactamase and exhibit multi-drug resistance (MDR) poses a substantial global threat, contributing to widespread foodborne illnesses and presenting an alarming issue for public health. This study specifically concentrated on the isolation and identification of ESBL-resistant genes (*bla*_TEM,_*bla*_SHV,_*bla*_CTX-M1,_*bla*_CTX-M2,_*bla*_CTX-M9,_*MultiCase*_ACC,_*MultiCase*_MOX,_*MultiCase*_DHA,_*bla*_OXA_) and the antibiogram profiling of *Salmonella enterica* serovars found in goat meat samples procured from retail outlets in Bangladesh. During the research in the Sylhet district of Bangladesh, researchers gathered a total of 210 samples of goat meat from 13 different Upazilas. Primarily, cultural and biochemical methods were used for isolation of bacteria from the selected samples. *Salmonella enterica* serovars Typhimurium and Enteritidis, along with three ESBL-resistant genes, were identified through polymerase chain reactions (PCRs). The disk diffusion test was used to determine antimicrobial susceptibilities. Out of 210 samples analysed, *Salmonella* spp*.* was detected in 18.10 % (38 out of 210), with *S.* Enteritidis and *S.* Typhimurium found in 9.05 % (19 out of 210) and 5.24 % (11 out of 210) of the samples, respectively. A total of 72.73 % (8/11) of *S.* Enteritidis and 100 % (19/19) of *S.* Typhimurium isolates were positive by Multidrug-resistant patterns. The positive outcomes were found of *S.* Typhimurium tested 63.16 % (12 out of 19) for the *bla*_TEM_ gene and 21.05 % (4/19) for the *bla*_SHV,_ gene. The study proposes that the retail goat meat market channel could be a prominent transmission way of ESBL-producing MDR *Salmonella enterica* serovars, representing a significant public health hazard.

## Highlights

• ESBL-producing multidrug-resistant Salmonella strains were alarmingly prevalent in retail goat meat across diverse regions of Bangladesh.
• Molecular analysis identified prominent ESBL gene *bla_TEM_* highlighting the genetic basis of antimicrobial resistance in Salmonella isolates from goat meat samples.
• Antimicrobial sensitivity profiling disclosed a worrisome resistance pattern, exacerbating the challenge of treatment options in cases of foodborne infections.
• Significant geographic correlations with antimicrobial resistance underscore the urgency for targeted surveillance and regulatory interventions to combat the spread of multidrug-resistant Salmonella in the food chain.

## Graphical abstract

**Figure:**
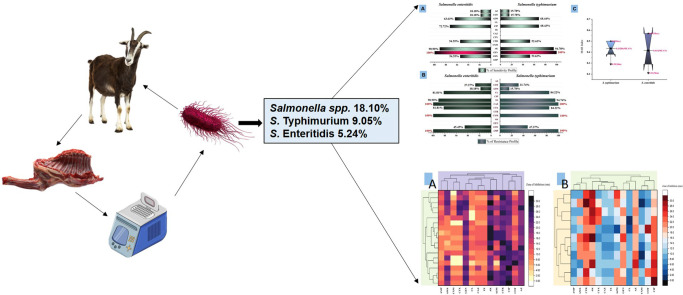

## 1 Introduction

The foodborne diseases (FBDs) are the major challenging approach for one health in the world. Annually an estimated 33 million lives are tragically dead due to foodborne infections, affecting one in ten individuals globally (World Health Organization, 2022). *Salmonella enterica*, a prevalent zoonotic foodborne pathogen, poses a significant obstacle to socioeconomic advancement, particularly in developing nations, impeding global progress (Hossain et al., 2021). In developed and developing countries the genus *S. enterica* is one of the most common causes of food-borne illness (Fàbrega & Vila, 2013). Notably, certain serovars like *S. Enteritidis, S. Typhimurium, S. Typhi*, and *S. Paratyphi*, contribute significantly to severe health complications in human populations (Jaja et al., 2019). The main source of *S. Enterica* transmission are animals based contaminated foods, which are responsible for foodborne diseases in humans (Li et al., 2021). Infection arises through the consumption of tainted poultry meat, goat meat and beef. The contamination of raw meat takes place at various stages of meat processing in butcher shops at retail markets (Hossain et al., 2021).

In Bangladesh, issues surrounding food contamination and adulteration are widely recognized as significant public health concerns. However, there remains a notable lack of understanding regarding consumer behaviour related to safe food purchasing (Science, 2020). The usual dietary intake in the nation predominantly features goat, cow, and poultry meat as the main protein sources (Uddin et al., 2019). Major meat safety concerns include inadequate hygiene practices during slaughter, consumption of undercooked meat, improper reheating of prepared meat at home and meat marketing, a significant prevalence of meat-borne diseases as *Salmonella* in humans, ineffective enforcement of animal slaughterhouse and meat inspection regulations, insufficient infrastructure, and a lack of awareness among various stakeholders (Bhandari et al., 2022). *Salmonella* is such a prevalent issue in food that effectively managing it poses significant challenges (Ehuwa et al., 2021). Various *Salmonella* serotypes have been identified in food-producing animals, with a significant number being regularly excreted in the feces of apparently healthy animals (Tagar & Qambrani, 2023).

Antibiotic resistance has emerged in food-producing animals as a result of the increased use of antibiotics in animal husbandry (Fatima et al., 2023). Regular and extensive antibiotic use in an environment creates a selective force that supports the survival of antibiotic-resistant bacteria, allowing them to enter the food chain (Fatima et al., 2023). Notably, contaminated *Salmonella* serotypes are renowned for disrupting the food chain integrity (World Health Organisation, 2022).The spread of multidrug-resistant (MDR) *Salmonella* through contaminated food consumption is a critical public health concern. Several studies have extensively documented the emergence of multidrug-resistant (MDR) Salmonella strains demonstrating resistance to essential antibiotics (Eng et al., 2015). Increasing acknowledgment of this phenomenon as a burgeoning global concern is becoming evident (Ejo et al., 2016; Hossain et al., 2021). Antimicrobial agents such as β-lactams and fluoroquinolones are frequently employed in the treatment of salmonellosis causing fowl typhoid (Zhang et al., 2022). On the other hand, the appearance of isolates of *Salmonella* that produce extended-spectrum β-lactamase (ESBL) poses a substantial risk, as it can result in therapeutic failure attributed to heightened resistance (Bush & Bradford, 2020).

Many developed countries utilize a systematic method to monitor and regulate antimicrobial resistance (AMR), functioning as a continuous surveillance system to track and manage AMR effectively (Adel et al., 2021). Conversely, in developing countries such as Bangladesh, the limited monitoring and surveillance systems are a result of inadequate surveillance networks, insufficient diagnostic precision, and underdeveloped laboratory capabilities (Fatima et al., 2023; Iskandar et al., 2021; Rabby et al., 2021). In recent years, numerous studies conducted in Bangladesh have focused on the detection and antibiogram profiling of Salmonella in retail poultry meat (Fatima et al., 2023; Rabby et al., 2021), but there is a scarcity of data concerning raw goat meat (Xu et al., 2020). Moreover, some investigations have documented the identification and frequency of resistance genes in *Salmonella* found in retail poultry meat in Bangladesh. Considering these facts, the current study investigated the ESBL producing MDR *Salmonella enterica* serovars in retail goat meat samples in Sylhet district of Bangladesh.

## 2 Materials and methods

### 2.1 Ethical consideration

The Animal Experimentation and Ethics Committee (AEEC) at Sylhet Agricultural University, Bangladesh, has thoroughly assessed and granted approval for the proposed experiment. The approved Animal Use Protocol is officially identified as #AUP2022034, outlining the ethical guidelines and procedures for the implementation of the experiment. This approval from the AEEC underscores the commitment to ensuring the humane treatment and welfare of the animals involved in the study. The rigorous evaluation conducted by the committee ensures that the experiment adheres to ethical standards, prioritizing the well-being and ethical treatment of the animals throughout the research process.

### 2.2 Research framework, geographic scope, and sampling approach

**Fig. 1:**
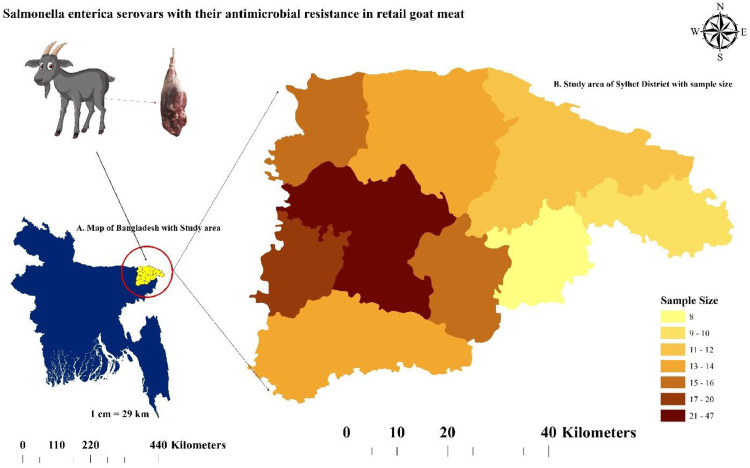
Study area map showing different upazila of Sylhet district with sample size. The choropleth map was created using ArcMap 10.7 (ESRI, USA).

A research investigation was conducted to examine various factors related to goat meat consumption in the Sylhet district of Bangladesh. The study was conducted in 13 Upazilas (sub-districts), specifically Balaganj, Beanibazar, Biswanath, Companiganj, Dakkhin Surma, Fenchuganj, Golapganj, Gowainghat, Jaintapur, Kanaighat, Osmaninagar, Sylhet Sadar, and Zakiganj. The geographical coordinates of these Upazilas were in the range of approximately 24°36′ to 25°11′ North latitude and 91°38′ to 92°30′ East longitude, as depicted in Fig. 1. To gather samples for the study, a "convenience sampling" approach was employed, taking into consideration the accessibility of retail goat meat shops in the respective areas.

#### 2.2.1 Determination of sample size

The required sample size for prevalence estimation was calculated using an equation described by (Mahen et al., 2024):

$$n=\frac{Z^{2}\mathrm{Pexp}\;\left(1-\mathrm{Pexp}\right)}{d^{2}}$$

Where, Pexp = expected prevalence, n = required sample size, d = desired absolute precision, Z = 1.96 for 95 % confidence interval level

Considering the previous data on the prevalence of *Salmonella* spp. in retail goat meat is 9 % (Naik et al., 2015) i.e., Pexp= 0.09 based on previous report was used to maximize the sample size. Using this Pexp with a desired absolute precision d=0.05, a required sample size of at least 126 were determined. This study was conducted with a total of 210 retail meat swab samples.

### 2.3 Collection of samples and isolation of bacterial strains

A total of 210 swab samples were procured from goat meat, sourced from diverse retail establishments across the Sylhet district. Swab samples were collected using aseptic techniques, ensuring thorough sterilization of equipment and personnel involved. Initially, pre-enriched retail meat swab samples were enhanced because all the samples were cultured in 225 ml of place buffered peptone water medium (BPW; Hi Media Laboratories Pvt. Ltd., Mumbai, India) in the ratio of 1:10 and incubated the medium at 37°C for 24 ± 2 h. Three drops of the pre-enriched culture from BPW then inoculated into MSRV enriched with 15 ml of Novobiocin Selective Supplement (Cat# SR0181E) and incubated at 42°C for 24 ± 3 h specifically for *Salmonella's* pre-enrichment. The pre-enriched culture from MSRV media was streaked onto *Salmonella Shigella* agar(SS) (Sigma-Aldrich, Germany) agar plates with loop full plates and incubated at 37°C for 24 ± 2 h (Mooijman, 2018). Colonies showing a single pinkish color with a black center were streaked onto xylose-lysine-deoxycholate (XLD) agar plates and incubated at 37°C for 24 ± 2 h for the selective enrichment of *Salmonella.* Positive *Salmonella* colonies were identified by their black-centered red color on the XLD agar (Oxoid, UK) plates. These positive colonies were then inoculated onto nutrient agar plates and incubated for 24 ± 3 h at 37 ± 1°C. Finally, positive colonies from the MacConkey agar (Oxoid, UK) plates were subcultured onto nutrient agar plates to obtain pure colonies and were incubated again for 24 ± 3 h at 37°C according to the procedures outlined in the ISO 6579 manual. Following culture and biochemical testing (including the sugar fermentation test, citrate test, methyl-red-Voges Proskauer test, motility, indole, and urease tests), presumptively identified *Salmonella* isolates were subjected to PCR for confirmation and detection.

### 2.4 Determination of *Salmonella enterica* serovars

The genomic DNA from *Salmonella* isolates was extracted following the manufacturer's guidelines, utilizing a DNA extraction kit (AddBio Inc. Ltd., Daejeon, Korea). To amplify the target genes of *Salmonella* spp., as well as those specific to the two predominant non-typhoidal *Salmonella enterica* serovars, Typhimurium and Enteritidis, uniplex PCR was carried out with three sets of reference primers. The primer sequences, along with other details, are provided in Table 1. The supplementary file (Supplementary file 1) outlines the required thermal cycle, temperatures, and durations for the PCR process. The genomic DNA from *Salmonella* isolates was extracted using a DNA extraction kit (AddBio Inc. Ltd., Daejeon, Korea), following the manufacturer's protocol. Bacterial DNA was extracted by boiling method with subsequent purification steps to ensure high-quality DNA (Hoque et al., 2023). To amplify the target genes specific to *Salmonella* spp., as well as those characteristic of the two predominant non-typhoidal *Salmonella enterica* serovars, Typhimurium and Enteritidis, uniplex PCR was conducted using three sets of reference primers. The primer sequences were selected based on their reported specificity and efficiency in previous studies (Supplementary file 1). Amplicon sizes for each target gene were provided in Table 1. Uniplex PCR was chosen over multiplex PCR to minimize potential primer interactions and optimize amplification conditions. However, it's worth noting that this approach may limit throughput and increase the required sample volume. Comprehensive details on the PCR cycling conditions, including annealing temperatures, extension times, and cycle numbers, are provided in the supplementary file (Supplementary file 1). This file ensures reproducibility and facilitates accurate replication of the PCR process.

**Table 1:** Primers used in PCR test of *Salmonella enterica* serovars and Beta-lactams resistant genes.

**Primer**	**Primer sequence**	**Product** **size**	**Target** **Organism/Genes**	**Reference**
*invA*	F- GTGAAATTATCGCCACGTTCGGGCAA R- TCATCGCACCGTCAAAGGAACC	284 bp	*Salmonella* spp.	(Dias De Oliveira et al., 2005)
*sefA*	F- GATACTGCTGAACGTAGAAGG R- GCGTAAATCAGCATCTGCAGTAGC	488 bp	*S*. Enteritidis	(Doran et al., 1996)
*fliC*	F- CGGTGTTGCCCAGGTTGGTAAT R- ACTGGTAAAGATGGCT	620 bp	*S.* Typhimurium	(Huen et al., 2019)
*bla*_TEM_	F- CATTTCCGTGTCGCCCTTATTC R- CGTTCATCCATAGTTGCCTGAC	800 bp	*TEM 1 & 2*	(Wang et al., 2019)
*bla*_SHV_	F- AGCCGCTTGAGCAAATTAAAC R- ATCCCGCAGATAAATCACCAC	713 bp	*SHV-1*	
*bla*_OXA_	F- GGCACCAGATTCAACTTTCAAG R- GACCCCAAGTTTCCTGTAAGTG	564 bp	*OXA-1,4 & 30*	
*bla*_CTX-M1_	F-TTAGGAAATGTGCCGCTGTA R-CGATATCGTTGGTGGTACCAT	688 bp	*CTX-M-1, CTX-M-3, & CTX-M-15*	
*bla*_CTX-M2_	F-CGTTAACGGCACGATGAC R-CGATATCGTTGGTGGTACCAT	404 bp	*CTX-M-2*	
*bla*_CTX-M9_	F-TCAAGCCTGCCGATCTGGT R-TGATTCTCGCCGCTGAAG	561 bp	*CTX-M-9 & CTX-M-14*	
*MultiCase*_ACC_	F-CACCTCCAGCGACTTGTTAC R-GTTAGCCAGCATCACGATCC	346 bp	*ACC-1 & ACC-2*	
*MultiCase*_MOX_	F-GCAACAACGACAATCCATCCT R-GGGATAGGCGTAACTCTCCCAA	895 bp	*MOX-1, MOX-2, CMY-1, CMY-8 to CMY-11& CMY-19*	
*MultiCase*_DHA_	F-TGATGGCACAGCAGGATATTC R-GCTTTGACTCTTTCGGTATTCG	997 bp	*DHA-1 & DHA-2*	

### 2.5 Antimicrobial susceptibility testing

The susceptibility of *Salmonella* isolates to antimicrobials was evaluated using the Kirby-Bauer disk diffusion method (Bauer et al., 1966; Skusa et al., 2022). Subsequently, the diameter of the inhibition zone was measured following the Clinical and Laboratory Standards Institute (CLSI, 2022) guidelines. A panel of 17 different antimicrobial agents, including ampicillin (AMP, 10 μg), amoxicillin (AMX, 10 μg), gentamicin (GEN, 10 μg), amikacin (AK, 30 μg), cefuroxime (CXM, 30 μg), ceftriaxone (CTR, 30 μg), cefotaxime (CTX, 30 μg), ceftazidime (CAZ, 30 μg), meropenem (MEM, 10 μg), imipenem (IMP, 10 μg), tetracycline (TE, 30 μg), ciprofloxacin (CIP, 5 μg), colistin (CST, 10 μg), azithromycin (AZM, 30 μg), chloramphenicol (CL, 30 μg), sulfamethoxazole-trimethoprim (COT, 1.25/23.75 μg), and nalidixic Acid (NA, 30 μg) was employed in the assay. The selection of these antibiotics was based on a comprehensive survey of over 100 prescriptions targeting Salmonella-related issues in goat farms, coupled with interviews of 100 veterinarians to identify commonly used antibiotics in salmonella infected farms. *Pseudomonas aeruginosa* ATCC 27853 was used for the quality control (QC) test in this study.

### 2.6 Molecular identification of extended-spectrum beta-lactamases (ESBLs) and ß-lactamase genes

Multiplex polymerase chain reaction (m-PCR) was utilized for the identification of resistance determinant genes – specifically *bla*_TEM,_ *bla*_SHV,_ *bla*_CTX-M1,_ *bla*_CTX-M2,_ *bla*_CTX-M9,_ *MultiCase*_ACC,_ *MultiCase*_MOX,_ *MultiCase*_DHA,_ *bla*_OXA_ in antibiotic-resistant isolates. The procedure employed specific oligonucleotide primers as detailed in Table 1 for targeted amplification of the genes of interest (Dallenne, da Costa, Decré, Favier, &Arlet, 2010). The PCR amplification protocols are depicted in Supplementary file-1.

### 2.7 Calculation of the multiple antibiotic resistance (MAR) index and identification of multi-drug resistance (MDR) status

The MAR index was computed and assessed based on the method outlined by (Mir et al., 2022)employing the formula: MAR= (The number of antibiotics to which an isolate was resistant) / (The total number of antibiotics tested)

If an isolate exhibited resistance to three or more classes of antimicrobials, it was classified MDR. MAR index values spanned from 0 to 1, with proximity to zero signifying high sensitivity and values nearing 1 indicating extreme resistance. A MAR index equal to or greater than 0.20 was considered indicative of a high-risk source for bacterial contamination or a state of significant "resistance."

### 2.8 Statistical analysis

The gathered data were systematically compiled, sorted, and structured within Excel spreadsheets. Disease prevalence rates were computed employing established formulas. Univariate analysis was performed using the Chi-square test to explore associations among different explanatory variables. Fisher's Exact Test was applied in instances where the expected count in a cell was less than 5 and occurred in at least 20 % of the cells (Hoque et al., 2023). Confidence intervals were determined using the Binomial exact test, with a significance level set at less than 0.05 to establish statistical significance. The entire data analysis process was executed using SPSS version 26.

### 2.9 Geo-spatial mapping and plot

The study area mapping was generated using ArcMap 10.7 (ArcMap 10.7, Esri USA), utilizing a shapefile extracted from (www.diva-gis.org). This data was employed to create choropleth, effectively visualizing the number of samples. Additionally, to illustrate the antimicrobial properties of isolates, we employed Origin-Pro (www.originlab.com) and utilized the Heat Map (Nahida Asha et al., 2024) and mirror bar diagram.

## 3 Results

### 3.1 Molecular prevalence

In this study, the overall prevalence of *Salmonella* spp*.* detected in retail goat meat was 18.10 % (38/210; 95 % CI: 13.13-23.98). Among the 13 upazilas in the Sylhet district, Jaintapur upazila exhibited the highest prevalence at 50.00 % (6/12), followed by Zakiganj with 40.00 % (4/10), while Golapganj upazila had the lowest prevalence at 6.25 % (1/16). The detailed distribution of *Salmonella* spp*.* prevalence in retail goat meat across different upazilas of Sylhet district were depicted in Table 2.

**Table 2:** Prevalence of *Salmonella* spp., *S.* Typhimurium *and S.* Enteritidis in accordance with different upazila of Sylhet District.

**Category**	***Salmonella* spp*.***			***Salmonella* Typhimurium**			***Salmonella* Enteritidis**		
	**n/N**	**Prevalence %**	***P-value***	**n/N**	**Prevalence %**	***P-value***	**n/N**	**Prevalence %**	***P-value***
**Location**			0.039			0.958			0.206
Sylhet Sadar	5/47	10.63 %		2/47	4.26 %		1/47	2.13 %	
Balaganj	3/14	21.43 %		1/14	7.14 %		1/14	7.14 %	
Beani Bazar	2/8	25.00 %		1/8	12.50 %		0/8	0 %	
Bishwanath	2/20	10.00 %		1/20	5.00 %		0/20	0 %	
Companiganj	1/15	6.67 %		0/15	0 %		1/15	6.67 %	
South Surma	2/17	11.76 %		1/17	5.88 %		0/17	0 %	
Fenchuganj	4/13	30.77 %		3/13	23.08 %		1/13	7.69 %	
Golapganj	1/16	6.25 %		1/16	6.25 %		2/16	12.50 %	
Gowainghat	2/14	14.28 %		1/14	7.14 %		0/14	0 %	
Jaintiapur	6/12	50.00 %		3/12	25.00 %		2/12	16.67 %	
Kanaighat	4/11	36.37 %		2/11	18.18 %		1/11	9.09 %	
Osmani Nagar	2/13	15.38 %		1/13	7.69 %		0/13	0 %	
Zakiganj	4/10	40.00 %		2/10	20.00 %		2/10	20.00 %	
**Total**	**38/210**	18.10 % (95 % CI: 13.13-23.98)		**19/210**	9.05 % (95 % CI: 5.54-13.77)		**11/210**	5.24 % (95 % CI: 2.64-9.18)	

N = Number of samples tested; n = Number of positive isolates; CI: Confidence interval

**Fig. 2:**
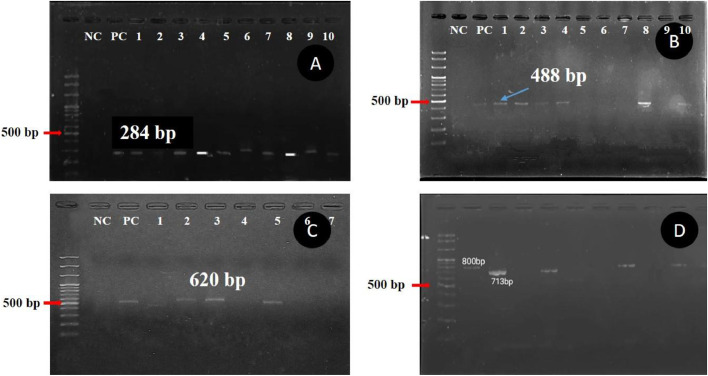
Amplified DNA of *invA* gene of *Salmonella* isolates at 284 bp, PC: Positive control as *Salmonella* spp. ATCC 700623 (Fig. 2A); *sefA* gene of *Salmonella* Enteritidis Isolated at 488 bp, PC: Positive control as *Salmonella enterica* ser. Enteritidis ATCC 13076 (Fig. 2B); *fliC* gene of *Salmonella* Typhimurium Isolated at 620 bp, PC: Positive control as *Salmonella enterica* ser. Typhimurium ATCC 700720 (Fig. 2C); NC: Negative control (Nuclease free water) Amplified DNA of ESBL Resistance genes of *Salmonella* spp. from first multiplex PCR test. Lane M: 100bp DNA Ladder, Lane M: 100bp DNA ladder, *bla_TEM_* gene at 800bp position. Lane 2: *bla_SHV_* gene at 713 bp (Fig. 2D).

The study revealed a prevalence of 9.05 % (95 % CI: 5.54-13.77) for *S.* Typhimurium and 5.24 % (95 % CI: 2.64-9.18) for *S.* Enteritidis (Fig. 2 and Table 2). Specifically, *S.* Typhimurium was most prevalent in Jaintapur district at 25 % (3/12), while no instances were detected in Companiganj Upazila. Conversely, *S.* Enteritidis was not detected in most upazilas (Beani bazar, Bishwanath, South Surma, Gowainghat and Osmaninagar) of Sylhet District, but the highest prevalence (20.00 %; 2/10) was observed in Zakiganj upazila within the Sylhet district (Table 2).

### 3.2 Characterization of antimicrobial resistance

#### 3.2.1 Phenotypic resistance pattern and MDR

Among the 11 *S.* Enteritidis isolates, five distinct phenotypic resistance patterns were identified. The most prevalent pattern (03) was characterized by resistance to eight antibiotics including AMP, AMX, CXM, CTX, CAZ, TE, CL, and COT from five different classes. Additionally, another pattern (AMP-CXM-CAZ) exhibited resistance to three antibiotics spanning two different classes. Notably, a substantial 72.73 % (8/11; 95 % CI: 39.03-93.98) of the *S.* Enteritidis isolates displayed multidrug resistance (Table 3). A total of seven distinct patterns were observed among the *S.* Typhimurium isolates (n = 19). The predominant pattern, observed in eight different isolates, exhibited resistance to seven antibiotics (AMP, AMX, CXM, CTX, CAZ, TE, CL). Strikingly, all 19 *S.* Typhimurium isolates demonstrated 100 % multidrug resistance (95 % CI: 82.35-100.00).

**Table 3:** Phenotypic resistant pattern of Multi-drug Resistant (MDR) *S.* Enteritidis and *S.* Typhimurium isolated from retail goat meat.

**Isolated Serovars**	**Phenotypic Pattern**	**No. of MDR phenotypic pattern**	**No. of Antibiotics**	**No. of Antimicrobial class**	**x/N; MDR % (95** % **CI)**
*S.* Enteritidis (n = 11)					8/11; 72.73 % (39.03-93.98)
	AMP-AMX-CXM-CTX-CAZ-TE-CL-AZM	2	8	5	
	AMP-AMX-CXM-CTX-CAZ-TE-CL-COT	3	8	5	
	AMP-CXM-CTX-CAZ-TE-CL	2	6	4	
	AMP-CXM-CAZ-TE	1	4	3	
	AMP-CXM-CAZ	3	3	2	
*S.* Typhimurium (n = 19)					
	AMP-AMX-CXM-CTX-CAZ-CL	1	6	3	19/19; 100.00 % (82.35-100.00)
	AMP-AMX-CXM-CTX-CAZ-TE-CL	8	7	4	
	AMP-CXM-CTX-CAZ-TE-CL	2	6	4	
	AMP-CXM-CTX-CAZ-TE-CL-COT	3	7	5	
	AMP-CXM-CTX-CAZ-TE-CL-AZM	2	7	5	
	AMP-CXM-CAZ-TE-AZM	1	5	4	
	AMP-CXM-CAZ-TE	2	4	3	
	**Total MDR isolates (%, 95** % **CI)**	**27/30 (90.00** %**, 73.47-97.89)**			

MDR = Multidrug Resistant; x/N: No. of MDR isolates/ Total No. of Isolates tested; CTX = cefotaxime; TE = tetracycline; AZM = azithromycin; COT = sulfamethoxazole-trimethoprim; CXM = cefuroxime; CAZ = ceftazidime; AMX = amoxicillin; AMP = ampicillin; CXM = cefuroxime; CL = colistin

#### 3.2.2 Antibiogram profiling and MAR index

**Fig. 3:**
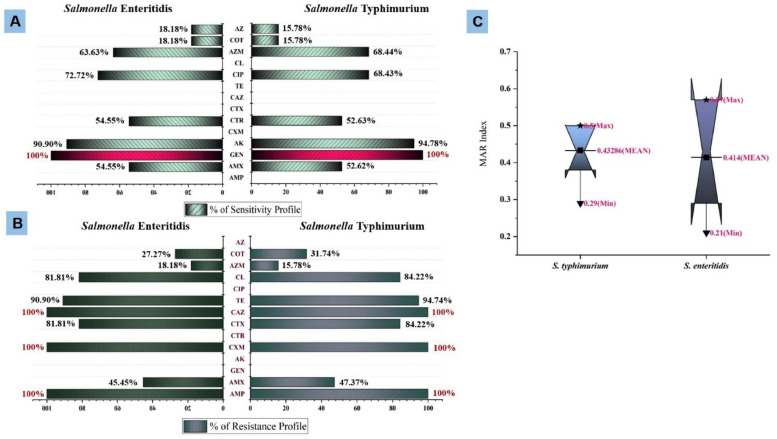
Antibiogram profiling of *Salmonella enterica* serovars isolated from retail goat meat. Fig. 3A showing the % of sensitivity profile; Fig. 3B showing % of resistance profile; Fig. 3C showing multiple antibiotic resistance index (MARI) of isolated serovars (*S.* Typhimurium and *S.* Enteritidis). CTX = cefotaxime; TE = tetracycline; AZM = azithromycin; COT = sulfamethoxazole-trimethoprim; CXM = cefuroxime; CAZ = ceftazidime; AMX = amoxicillin; AMP = ampicillin; CXM = cefuroxime; CL = Colistin, GEN = gentamicin; AK = amikacin; CTR = ceftriaxone; CIP = ciprofloxacin

This study involved subjecting all isolates of *S. enterica* serovars to antibiotic sensitivity profiling using 14 antibiotic disks. Among the 30 *S. enterica* serovar isolates, the CLSI standard revealed 100 % resistance to ampicillin, cefuroxime, and ceftazidime. Notably, no resistance was observed for gentamicin, amikacin, and ciprofloxacin, as detailed in Fig. 3. Regarding the sensitivity profiles, *Salmonella* Typhimurium isolates exhibited 100 % sensitive to gentamicin and 94.78 % sensitive to amikacin. Similarly, *S.* Enteritidis displayed 90.90 % sensitive to amikacin and 100 % sensitivity to gentamicin.

**Fig. 4:**
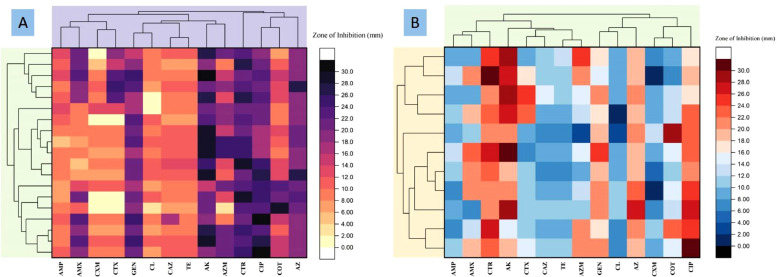
Heatmap with dendogram showing the actual antimicrobial sensitivity profile (Zone of inhibition in mm) and the dendogram showing the correlation (Pearson's correlation) among the isolated sample in response to their inhibitory activity. Light yellowish indicates extremely resistant whereas deep purple indicates extremely sensitive (Fig. 4A; *S*. Typhimurium); Deep sky bluewish to light indicates extremely resistance to antibiotics (Fig. 4B; *S.* Enteritidis). CTX = cefotaxime; TE = cetracycline; AZM = azithromycin; COT = sulfamethoxazole-trimethoprim; CXM = cefuroxime; CAZ = ceftazidime; AMX = amoxicillin; AMP = ampicillin; CXM = cefuroxime; CL = colistin, GEN = gentamicin; AK = amikacin; CTR = ceftriaxone; CIP = ciprofloxacin

Fig. 4A presents a heatmap with a dendrogram, illustrating the actual zone of inhibition in millimeters (mm) for isolated samples of *S.* Typhimurium. The color gradient ranges from light yellowish, indicating extreme resistance, to deep purple, indicating extreme sensitivity. Additionally, the dendrogram illustrates the Pearson's correlation, depicting the relationship among isolated samples based on their inhibitory activity. In Fig. 4B, a similar heatmap with a dendrogram showcases the inhibitory activity of isolated samples of *S.* Enteritidis. The color spectrum, ranging from deep sky blue to light, represents varying degrees of resistance to antibiotics, with deep indicating extreme resistance.

In Fig. 3C, the multiple antibiotic resistance index (MARI) values for *S.* Typhimurium ranged from 0.29 to 0.50, with an average index of 0.43. In contrast, for *S.* Enteritidis, the MARI values spanned from 0.21 to 0.57, averaging at 0.41.

### 3.3 Detection of ESBL resistance

Only two of the nine tested extended-spectrum beta-lactamase (ESBL) resistance genes were detected among the *S. enterica* serovars in the study (Table 4). Significantly higher frequencies of ESBL resistance, specifically the *bla*_TEM_genes, were observed in both *S.* Typhimurium and *S.* Enteritidis isolates (P<0.001). The prevalence of *bla*_TEM_ was 63.16 % for *S.* Typhimurium and 36.36 % for *S.* Enteritidis. The resistance index (RI) for the positive ESBL resistance gene was 0.22 (2 out of 9) for both isolates.

**Table 4:** Frequency of beta-lactams resistance genes in *Salmonella* Enteritidis *and* Typhimurium isolated from retail goat meat.

***Salmonella enterica* serovars**	**β lactams Resistant genes**	**No. of total isolates**	**No. of positive isolates**	**Frequency % (95** % **CI)**	**P-value**	**RI**
*Salmonella* Typhimurium (n = 19)					<0.001	0.22
	*bla*_TEM_	19	12	63.16 % (38.36-83.71)		
	*bla*_SHV_	19	4	21.05 % (6.05-45.57)		
*Salmonella* Enteritidis (n = 11)					<0.001	0.22
	*bla*_TEM_	11	4	36.36 % (10.93-69.21)		
	*bla*_SHV_	11	1	9.09 % (0.23-41.28)		

CI: Confidence interval; RI: Resistance Index

## 4 Discussion

*Salmonella enterica* serovars is a leading cause of human foodborne bacterial gastroenteritis (Gong et al., 2016). The overall prevalence rate of *Salmonella* spp. in retail goat meat was found to be 18.10 % in this study, which is higher to the previous studies where the prevalence of *Salmonella* from goat carcass swab was 9 % at India,8.3 % at Modjo and 7.5 % at Bishoftu, 4 % at Arusha (Tanzania), 3.5 % at Gujrat in India (Gaspary et al., 2016; Makwana et al., 2015; Naik et al., 2015; Woldemariam et al., 2005). The high prevalence might be related to the wet market collecting of meat samples from goat slaughtered in unsanitary settings and eviscerated in contaminated regions with intestinal contents. In the majority of cases, no specific slaughterhouse was identified in the retail meat market. Instead, meat shop traders commonly slaughter goats either in front of or behind their shops, increasing the likelihood of meat contamination with environmental pathogens. In the Sylhet district of Bangladesh, a noteworthy practice involves selling animal intestines in the same section as the meat, presenting a significant source of gut pathogens such as *Salmonella*.

In this study, the prevalence varied from place to place. The highest prevalence (50 %) was found in Jaintiapur in compare to other places. However, the lowest prevalence was 6.67 % in Companiganj. The reason of variation might be due to retail shop hygiene, shop keeper knowledge related to contamination, space of shop, urban and rural area.

*Salmonella* spp. isolated from separate retail goat meat sample contained *S.* Typhimurium and *S.* Enteritidis. In this study, nineteen (9.04 %) isolates detected as *S.* Typhimurium, while eleven (5.23 %), isolates were detected as *S.* Enteritidis which is more than to the study reported by Makwana et al., (2015) in Goat meat. Makwana et al., (2015) found the prevalence of *S.* Typhimurium (3.87 %; 11/284) and *S.* Enteritidis (0.70 %; 2/284) in Gujrat, India. *Salmonella* Typhimurium emerged as the predominant serovar in the conducted study. In recent decades, *S.* Typhimurium and *S.* Enteritidis have become significant contributors to foodborne salmonellosis on a global scale (Thung et al., 2019). Most *Salmonella* infections have been associated with the consumption of contaminated chicken, pork, and beef products, with *S.* Enteritidis and *S.* Typhimurium being the most frequently identified isolates (Yang et al., 2015). An increasing rate of antimicrobial resistance in *Salmonella* has been reported in many developing and developed countries (Ashtiani et al., 2009) and resistance to combinations of several classes of antimicrobials has led to the emergence of MDR strains. These antibiotics are expected to be widely used in Bangladesh's goat farming system because to highly resistance of *S.* Typhimurium and *S.* Enteritidis to ampicillin, cefuroxime, ceftaxime, tetracycline and colistin in our study. But there is research analyzed by Saha et al., (2014), who found isolated *Salmonella* spp. showed various degrees of sensitivity to oxytetracycline, gentamycin, sulphamethoxazole, spiramycin, streptomycin, amoxicillin, penicillin-G, and ciprofloxacin. Resistance of MDR *Salmonella* to ampicillin, amoxicillin, cefuroxime, cefotaxime, tetracycline and colistin is increasing significantly which pose a threat to the public health (Ashtiani et al., 2009). In this research, *Salmonella* Typhimurium is 100 % resistant to tetracycline, ceftazidime, cefotaxime and ampicillin.

Gram-negative Enterobacteriaceae, such as *Salmonella* spp, *Klebsiella pneumoniae*, and *E. coli* are the primary producers of ESBLs (Khadka et al., 2023). In bacteria, the synthesis of B- lactamases is thought to be the principal mechanism of resistance to B-lactam antibiotics. ESBLs are often encoded by large plasmids that can be passed from one bacterial species to the next (Khadka et al., 2023). The most common types of β-lactamases are classified into the following groups: Resistance to first and second generation cephalosporins is acquired by *TEM-1* and *OXA-1*; Cephamycin resistance is generated by AmpC-Lactamase (CMY) and resistance to broad-spectrum cephalosporins is conferred by ESBL (Husna et al., 2023). In this study we tried to detect ESBL producing *TEM-1 &2, OXA-1, 4 & 30, CTX-M-1, CTX-M-3, CTX-M-15, CTX-M-2, CTX-M-9 & CTX-M-14, ACC-1 & ACC-2, MOX-1, MOX-2, CMY-1, CMY-8, CMY-19, and DHA-1 & DHA-2* genes. But in this research, we got *bla*_TEM_*, bla*_SHV_ ESBL producing genes for both *S.* Typhimurium and *S.* Enteritidis. In this investigation, the ESBL resistance genes *bla*_TEM_*, bla*_SHV_ were discovered to be positive in *Salmonella enterica* serovars, with prevalence of 63.05 %, 21.05 % respectively and the other genes were completely absent. The current findings showed an extremely high the frequency of *bla*_TEM_ in *S.* Typhimurium. Similar findings were seen in case of retail meat in Egypt by Adel et al., (2021) .

Our findings also suggest that wet markets that handle goat meat serve as reservoirs for *Salmonella enterica* serovars. Understanding the need for *Salmonella* control during slaughter is crucial, as cross-contamination may occur, potentially contaminating the goat carcass with multidrug-resistant *Salmonella enterica* serovars. MDR *Salmonella enterica* serovars pose a risk to humans as well. To lower the incidence of food-borne disease at retail goat meat markets, more appropriate intervention techniques, such as cleaning, drainage, waste management, awareness, and training, are required. This study has explored a severe public health risk in this region, underscoring the need for strong monitoring, policy implementation, and public awareness campaigns to combat the health hazards posed by salmonellosis.

## 5 Conclusion

In the present study, pathogenic MDR *Salmonella enterica* serovars carried by retail goat meat were detected in goat meat retail markets. The goat meat market might be regarded as a source for the transmission and infection of MDR *Salmonella enterica* serovars, which can readily attach to vendors, customers, and the food chain due to inadequate sanitation and hygiene practices. Additionally, the prevalence of resistance genes in goat meat emphasizes the necessity of focused interventions. Subsequent investigations should prioritize to understand the mechanisms underlying antibiotic resistance in *Salmonella enterica* serovars and formulating efficient approaches to mitigate multidrug resistance and ESBL production within the food chain. Collaborative efforts involving public health authorities, veterinarians, food producers, and consumers are imperative to tackle this pressing public health concern and curtail the proliferation of antibiotic-resistant bacteria throughout the food supply chain.

## Financial disclosure

This study received financial support from the Sylhet Agricultural University Research System (SAURES) under the auspices of University Grant Commission (UGC) of Bangladesh.

## Ethical statement

The Animal Experimentation and Ethics Committee (AEEC) at Sylhet Agricultural University, Bangladesh, has thoroughly assessed and granted approval for the proposed experiment. The approved Animal Use Protocol is officially identified as #AUP2022034, outlining the ethical guidelines and procedures for the implementation of the experiment. This approval from the AEEC underscores the commitment to ensuring the humane treatment and welfare of the animals involved in the study. The rigorous evaluation conducted by the committee ensures that the experiment adheres to ethical standards, prioritizing the well-being and ethical treatment of the animals throughout the research process.

## CRediT authorship contribution statement

**Jarin Al Naser:** Writing – review & editing, Writing – original draft, Visualization, Validation, Software, Resources, Methodology, Investigation, Formal analysis, Data curation. **Hemayet Hossain:** Writing – original draft, Visualization, Validation, Software, Resources, Methodology, Investigation, Formal analysis, Data curation. **Md. Shahidur Rahman Chowdhury:** Writing – original draft, Visualization, Validation, Software, Resources, Methodology, Investigation, Formal analysis, Data curation. **Nasrin Akter Liza:** Writing – original draft, Visualization, Validation, Supervision, Software, Resources, Methodology, Investigation, Formal analysis, Data curation. **Rayhan Mahmud Lasker:** Writing – original draft, Visualization, Validation, Software, Resources, Methodology, Investigation, Formal analysis, Data curation. **Asikur Rahman:** Writing – original draft, Visualization, Validation, Software, Resources, Investigation, Formal analysis, Data curation. **Md. Ariful Haque:** Writing – original draft, Visualization, Validation, Software, Resources, Methodology, Investigation, Formal analysis, Data curation. **Md. Mukter Hossain:** Writing – original draft, Visualization, Validation, Supervision, Software, Resources, Methodology, Investigation, Formal analysis, Data curation. **Md. Mahfujur Rahman:** .

## Declaration of competing interest

The authors declare the following financial interests/personal relationships which may be considered as potential competing interests: Md. Mahfujur Rahman reports financial support was provided by Sylhet Agricultural University Research System (SAURES). If there are other authors, they declare that they have no known competing financial interests or personal relationships that could have appeared to influence the work reported in this paper.

## Data Availability

The data supporting the findings of this study are available from the corresponding author upon reasonable request.

## Appendix Supplementary materials

Image, application 1
