# Supplementary material for: Exploring of spectrum beta lactamase producing multidrug-resistant Salmonella enterica serovars in goat meat markets of Bangladesh
Source: Vet Anim Sci. 2024 Jun 2;25:100367. doi: 10.1016/j.vas.2024.100367 (PMC11214345; doi:10.1016/j.vas.2024.100367)
Supplement: Supplementary file 1 [file mmc1.docx]

**Supplementary Table 1:** Thermal cycle for PCR testing for identifying Salmonella spp., *S.* typhimurium and *S.* enteritidis using 3 different primers set

| **Primer** | **Thermal Cycle** | | | | **Temperature and Time** | **Reference** |
| --- | --- | --- | --- | --- | --- | --- |
| invA | Initial Denaturation | | | | 95℃, 1 min | (Oliveira et al., 2003) |
|  | Denaturation  Annealing  Extention | (35 cycles) | | | 95℃,30 sec  64℃,30 sec  72℃,30sec |  |
|  | Final Extention | | | | 72℃,4min |  |
|  | Hold | | | | ∞ |  |
| sefA | Initial Denaturation | | | | 95℃, 5 min | (Doran et al., 1996) |
|  | Denaturation  Annealing  Extention | | (35 cycles) | | 94℃,1 min  55℃,1 min  72℃,1 min |  |
|  | Final Extention | | | | 77℃, 7 min |  |
|  | Hold | | | | ∞ |  |
| fliC | Initial Denaturation | | | | 94℃, 5 min | (Soumet et al., 1999) |
|  | Denaturation  Annealing  Extention | | | (35 cycles) | 94℃,1 sec  55℃,1 sec  72℃,21sec |  |
|  | Final Extention | | | | 72℃, 7min |  |
|  | Hold | | | | ∞ |  |

**Supplementary Table 2:** Thermal cycle for mPCR of *ESBL genes*

| **Sl No.** | **Steps** | **Temperature(℃)** | **Time** | **Cycle** | **Reference** |
| --- | --- | --- | --- | --- | --- |
| **1** | Initial Denaturation | 95℃ | 5min | 1 | (Bobbadi et al., 2020) |
| **2** | Final Denaturation | 94℃ | 40sec | 30 |  |
| **3** | Anneling | 60℃ | 40sec |  |  |
| **4** | Initial Extension | 72℃ | 1min |  |  |
| **5** | Final Extension | 72℃ | 7min | 1 |  |
